# Continuous Purification of Colloidal Quantum Dots in Large-Scale Using Porous Electrodes in Flow Channel

**DOI:** 10.1038/srep43581

**Published:** 2017-02-27

**Authors:** Hosub Lim, Ju Young Woo, Doh C. Lee, Jinkee Lee, Sohee Jeong, Duckjong Kim

**Affiliations:** 1 Department of Nano Mechanics, Korea Institute of Machinery and Materials (KIMM), Daejeon 34103, Republic of Korea; 2 School of Mechanical Engineering, Sungkyunkwan University, Suwon, Gyeonggi-do 16419, Republic of Korea; 3 Department of Chemical and Biomolecular Engineering (BK21+Program), KAIST Institute for the Nanocentury, Korea Advanced Institute of Science and Technology (KAIST), Daejeon 34141, Republic of Korea; 4 University of Science and Technology (UST), 217 Gajeong-ro, Daejeon 34113, Republic of Korea

## Abstract

Colloidal quantum dots (QDs) afford huge potential in numerous applications owing to their excellent optical and electronic properties. After the synthesis of QDs, separating QDs from unreacted impurities in large scale is one of the biggest issues to achieve scalable and high performance optoelectronic applications. Thus far, however, continuous purification method, which is essential for mass production, has rarely been reported. In this study, we developed a new continuous purification process that is suitable to the mass production of high-quality QDs. As-synthesized QDs are driven by electrophoresis in a flow channel and captured by porous electrodes and finally separated from the unreacted impurities. Nuclear magnetic resonance and ultraviolet/visible/near-infrared absorption spectroscopic data clearly showed that the impurities were efficiently removed from QDs with the purification yield, defined as the ratio of the mass of purified QDs to that of QDs in the crude solution, up to 87%. Also, we could successfully predict the purification yield depending on purification conditions with a simple theoretical model. The proposed large-scale purification process could be an important cornerstone for the mass production and industrial use of high-quality QDs.

Colloidal quantum dots (QDs) have gained immense attention because of their potentials in many applications such as displays, lighting, bio-imaging, photocatalysis, and photovoltaics^1^,^2^,^3^,^4^,^5^. QDs are typically synthesized in solution with a large number of hydrocarbons that allows high-temperature crystal growth^6^,^7^. After the synthesis, unreacted precursors, excess amounts of surfactants, and reaction media need to be effectively removed from the QDs since such impurities likely to degrade the performance of QD-based optoelectronic devices^8^,^9^,^10^. For the purification of QDs, the precipitation–dissolution method which is followed by centrifugation is the most commonly used technique^11^,^12^,^13^. Relatively polar solvents such as alcohol and acetone are added as non-solvents to form QD aggregates by decreasing solubility of QDs. Resulting QD aggregates are usually collected by centrifugation and finally redispersed in target organic solvents. The precipitation and redispersion processes are repeated several times until the impurities are sufficiently removed^6^. This conventional technique requires expensive centrifuges and large amounts of solvents; it is also not scalable or controllable. Moreover, the efficiency of this technique depends on the precursor and the size and morphology of the QDs which is very complicated to establish a theoretical model^14^,^15^,^16^. Recently, the large-scale synthesis of QDs has been presented owing to their various usage in the industry. It has been reported that the continuous synthesis process could be scaled up^17^,^18^,^19^,^20^. However, without developing continuous large scale purification together, mass production of QDs still remains challenging. Electrophoretic deposition (EPD) method has been used to assemble the nanocrystals on various substrates and it was demonstrated that the method can be used for solar cell applications^21^,^22^,^23^. Using EPD, Bass *et al*. developed a quick and efficient technique for purification of QDs but it was still a batch-type process which is not suitable for mass purification^24^. To improve this method, our group presented a continuous QD purification process based on microfluidics and electrophoresis^25^. The fluid flow is perpendicular to electrical field limiting the purification yield due to the lack of retention time of QDs exposed to the electric field.

In this work, we developed a new continuous purification process that is adaptable to the mass production of high-quality QDs. In this process, the as-synthesized QD dispersion solution is infused into a flow channel with porous electrodes. When an electric field is applied between adjacent electrodes, the QDs are collected on the porous electrodes while unreacted impurities pass through the electrodes. Nuclear magnetic resonance (NMR) analysis and ultraviolet/visible/near-infrared (UV/Vis/NIR) absorption spectroscopy clearly revealed that impurities in collected QDs are dramatically reduced. The purification yield of our process was calculated based on the intensity change of the first exciton peak in the absorption spectrum. To describe the purification process, we built a simplified analytical model for the movement of QDs driven by fluid flow and electrophoresis. Finally, we estimated the surface area of porous electrodes required for the purification of specified amounts of QDs and discuss the scalability of the proposed process.

## Results and Discussion

### Electrophoretic QD purification device with porous electrodes

**Figure 1:**
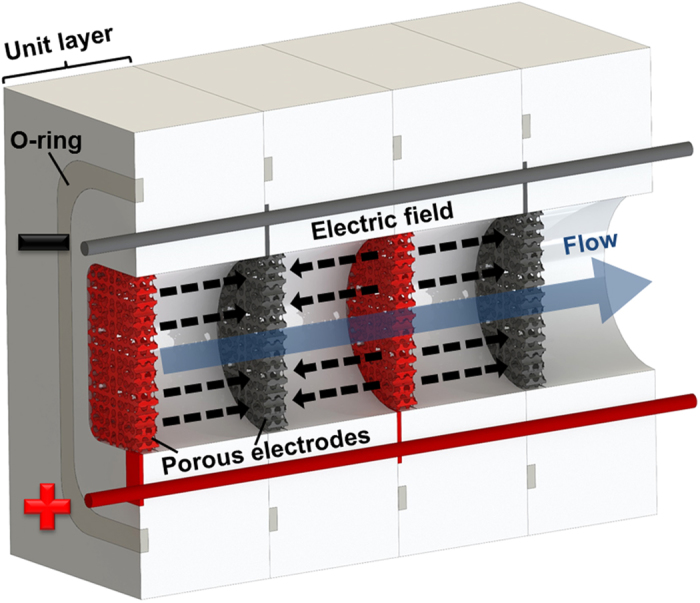
Schematic diagram of the purification system with polytetrafluoroethylene unit layer, O-ring, and porous nickel electrodes.

Electrophoresis is the method used to manipulate micro- or nano-sized particles using an electric field^26^. The electrophoretic behavior of particles is relatively well understood and highly controllable. Here, we used a flow channel system with porous electrodes to capture QDs from a QD dispersion solution passing through the system by using electrophoresis, as shown in Fig. 1. The dashed lines represent the electric field between the electrodes with positive (red color) and negative (gray color) electric potential and the blue arrow indicates the flow direction. As shown in Fig. 1, the flow direction is parallel to the electric field, in contrast to our previous work^25^. To design the new system, a particle-tracing simulation was performed, which showed that QDs can be more effectively captured by the electrodes when the flow direction is parallel to the electric field (Figures S1 and S2). Moreover, in the new process, the captured QDs can be thoroughly washed to remove impurities whereas our previous work did not include a washing step.

The proposed electrophoretic purification device is operated in three steps. First, when the electric field is applied between the electrodes, the QDs migrate toward the electrodes and adhere onto their surfaces (Figure S3). Then, a non-solvent solution (acetone and ethanol) flows through the channel to wash out impurities and induces aggregation of the nanoparticles on the electrode surface. Finally, the fresh solvent (toluene) is applied to redisperse the nanoparticles after removing the impurities.

**Figure 2:**
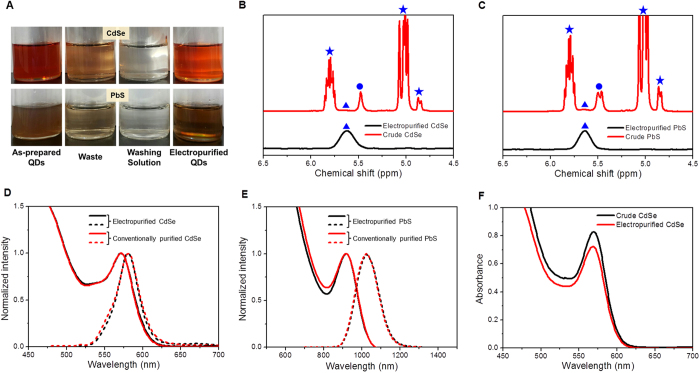
Solution images, ^1^H NMR spectra, emission and absorbance of QDs. (**A**) Photographs of materials (as-prepared QDs, waste, washing solution, and electropurified QDs) (**B**) ^1^H NMR spectra of crude and electropurified CdSe. (**C**) ^1^H NMR spectra of crude and electropurified PbS. The resonances can be attributed to octadecene (★), free oleate (•), metal oleate (•), and bound oleate (▴). (**D**) Absorption (solid line) and emission (dotted line) spectra of CdSe QDs. (**E**) Absorption (solid line) and emission (dotted line) spectra of PbS QDs. (**F**) CdSe absorption peak (electropurified QDs and crude QDs) used to calculate the purification yield.

To conduct the purification experiment, CdSe and PbS QDs were prepared following previously reported methods (see Materials and Methods for details). The crude QD solution contained the primary solvent (octadecene) and contaminants, such as excess oleic acid (OA), leftover precursors (cadmium oleate), and reaction byproducts. The crude solution was mixed with toluene to improve its stability and with non-solvent (acetone and ethanol) to enhance its electrophoretic mobility. Figure 2A shows the as-prepared solution and three other solutions, the waste solution, washing solution, and purified QD solution, after the purification process. The waste solution included impurities and QDs that did not adhere onto the electrode surfaces and the as-prepared solution was flown through the channel upon the application of the electric field. The washing solution was a mixture of acetone and ethanol used to wash the adsorbed QDs. The purified QD solution was the nonpolar solvent containing the purified QDs.

The electropurified QDs were analyzed by ^1^H NMR spectroscopy, which can distinguish surface-bound ligands and impurities (*e.g*., free OA, metal oleate, and organic solvent) to determine the elimination of impurities^27^,^28^,^29^,^30^. As shown in Fig. 2B,C, the electropurified QDs contained surface-bound OA (triangles) nearly without impurities, in contrast to the QD crude solution, which included a large amount of impurities, including octadecene (asterisks) and free and metal-ligand complexes (sphere). For a comparison with electrophoretic purification, the chemical components of conventionally purified QDs were also measured using NMR. These measurements revealed that the precipitation–redispersion process needed to be repeated at least twice to remove all the impurities (see Figure S4). Moreover, 50 mL of non-solvent were typically required to purify 5 mL of QD solution (see Materials and Methods for details), which is approximately 10 times higher than the quantity required in the electrophoretic purification method. We note that QD charging effect, which has been known to significantly quench the PL of QDs, was almost negligible for electropurified QDs, showing higher relative PL compared to conventionally purified QDs (see Figure S5)^31^. We also checked if redox reaction occurs due to the electric field in the course of the electrophoretic purification by using X-ray photoelectron spectroscopy (XPS) analysis. The XPS spectra show that there is not any sign on the formation of oxidized species for electropurified QDs regardless of the applied voltage (see Figure S6).

A comparison was performed between the electropurified QDs and the conventionally purified QDs using CdSe and PbS nanoparticles. Figures 2D and 2E show the absorption (solid lines) and emission (dotted lines) spectra of electropurified and conventionally purified CdSe and PbS QDs. The positions of the first exciton peaks in the absorption and emission spectra were nearly identical: 572 nm for the absorption and 581 nm for the emission in the case of CdSe and 917 nm for the absorption and 1023 nm for the emission in the case of PbS. This agreement in the first exciton peak positions indicates there was no significant variation in the optical properties of the purified QDs regardless of the purification method. UV/Vis/NIR absorption spectroscopy also confirmed the good purification results for CdSe and PbS QDs. The detailed results are available in Supporting Information (see Figure S7).

To calculate the purification yield of the electrophoretic purification, we used UV/Vis/NIR absorption spectra acquired before and after the purification (Fig. 2F). The purification yield was defined as the ratio of the mass of purified QDs to that of QDs in the crude solution. The QD concentration was calculated using the absorbance of the first exciton peak in the absorption spectra divided by the extinction coefficient of the QDs and the optical path length considered in the measurement, according to the Beer-Lambert law (see Figure S8). We obtained the mass of the QDs by multiplying the QD concentration with the total solution volume. Because the extinction coefficient, the path length, and the solution volume were the same for both solutions, the purification yield was equal to the ratio of the first exciton peak of the purified QDs to that of the crude QDs. When the non-solvent content was 70%, the intensity of the first exciton peak of the as-prepared QD solution was approximately 0.828 and that of the purified QD solution was about 0.722, as shown in Fig. 2F. In this case, the purification yield was calculated to be 87.2%.

### Purification yield estimation model

**Figure 3:**
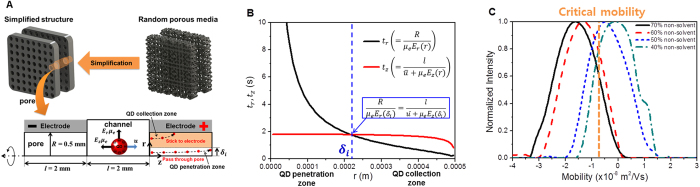
Purification yield estimation model. (**A**) Simple model describing the QD purification process and (**B**) estimated QD travel time. (**C**) Distributions of electrophoretic mobility of CdSe QDs.

To understand the purification process, we built a simple model using the movement of QDs driven by the fluid flow and electrophoresis. Figure 3A shows a schematic of the simplified porous electrodes that were used for the simulation. A porous electrode with a random pore network was simplified as a planar electrode with an array of circular holes and a 2 mm gap between the electrodes. The hole diameter and distance between the holes were determined based on the pore geometry of the electrode. The radius of the hole (*R*) was 0.5 mm and the length of the electrode (*l*) was 2 mm. In this model, the electric potential of the right electrode was positive and that on the left electrode was negative, similarly with the experiment. Because QDs with negative electrophoretic mobility adhere to the positive electrode, we focused on the movement of QDs near the entrance of the positive electrode. The hole in the positive electrode can be divided into two areas with respect to the movement of QDs. The orange-colored area represents the QD collection zone and QDs in this zone eventually attach onto the electrode; the other area is the QD penetration zone, where QDs pass through the hole without adhering onto the electrode. We assumed that QDs were uniformly distributed over the entrance of the positive electrode hole. The purification yield was calculated by the ratio of cross-sectional area of the QD collection zone to the inlet area, considering that each area is proportional to the amount of QDs; namely, the inlet area is proportional to the number of infused QDs and the cross-sectional area of the QD collection zone to the number of collected QDs. The travel times of the QDs in the r- and z-directions are as follows:


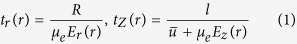


where *t*_*r*_ is the r-directional travel time of QDs reaching the electrode surface by electrophoresis, *t*_*z*_ is the z-directional travel time of QDs passing through the electrode by electrophoresis and fluid flow, *E*_*r*_ is the electric field strength in the r-direction, *E*_*z*_ is the electric field strength in the z-direction at the entrance of the positive electrode hole, and μ_e_ is the electrophoretic mobility of the QDs. The electric field strengths in the r- and z-directions were calculated using a numerical simulation with commercial software (COMSOL). The average velocity of the flow (*ū*) equals the flow rate divided by the number and area of the holes.

When *t*_*z*_ < *t*_*r*_, the QDs pass through the electrode before reaching the electrode surface. In contrast, when *t*_*z*_ > *t*_*r*_, the QDs are captured by the electrode because of the r-directional electrophoretic movement. Hence, the radius of the QD penetration zone (*δ*_*i*_) can be calculated when *t*_*z*_ = *t*_*r*_ as follows


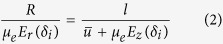


Figure 3B shows how *δ*_*i*_ is determined for a specified mobility of QDs. However, in real situations, the mobility has a Gaussian distribution; for example, Fig. 3C shows the electrical mobility of CdSe and *δ*_*i*_ must be calculated for each mobility value. The purification yield (*y*_*i*_) for each *δ*_*i*_ can be calculated as follows:


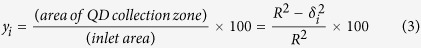


The total yield is calculated by the integration of yield function, *y*_*i*_(μ_*e*_), multiplied by the experimentally found electrophoretic mobility distribution function, *m*(μ_*e*_) as follows:


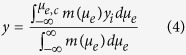


where μ_*e,c*_ is the critical mobility. In this integration, we included *y*_*i*_ only if the mobility was below the critical mobility decided from *m*(μ_*e*_) and experimental data on the purification yield, which was equivalent to the minimum zeta potential required for the adherence of QDs onto the electrode surface to overcome the physical hindrance caused by surface ligands.

### Comparison of experimental and analytical results

**Figure 4:**
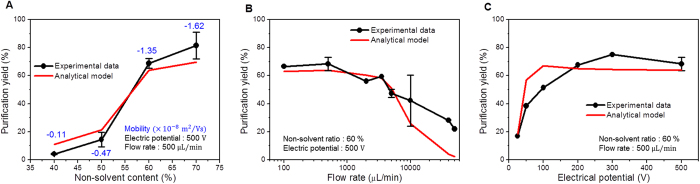
Theoretical estimation of purification yields. Purification yield determined by the experiments and the analytical model as a function of (**A**) non-solvent content, (**B**) flow rate, and (**C**) electric potential difference.

We investigated theoretically and experimentally how the purification yield was affected by the several process conditions, such as the electrophoretic mobility of the QDs, the flow rate, and electrical field. Figure 4A shows the purification yield for a non-solvent content ranging from 40 to 70%. The absolute value of the electrophoretic mobility of the QDs increases as the non-solvent content increases. Other experimental conditions were maintained stable, with a 500 V electrical potential and a 500 μL/min flow rate. Because the electrophoretic mobility of the nanoparticle directly affects the migration of the QDs in the electric field, more QDs of the as-prepared QD solution tend to adhere onto the electrode as the electrophoretic mobility increases, as shown in Fig. 4A. When the non-solvent content is above 60%, the purification yield dramatically increases to over 70%. This dramatic increase in the purification yield indicates the presence of a threshold electrophoretic mobility for the purification process; we refer to this threshold value as critical mobility. We determined that the critical mobility was −7.49 × 10^−9^ m^2^/Vs by fitting the experimental data with the analytical results.

When the flow rate of the as-prepared solution increases, the residence time during which the QDs are exposed to the electric field decreases and the shear force at the electrode surface increases. Then, the purification yield decreases as the flow rate increases(Fig. 4B). However, the purification yield almost reaches a plateau when the flow rate is lower than 500 μL/min. This indicates that the effect of the electrophoretic force on the movement of QDs is dominant in the low flow rate regime. The analytical results were in good agreement with the experimental data.

The electric potential difference also affects the purification yield of the QDs. Figure 4C shows the purification yield as a function of electrical potential for the same flow rate of as-prepared solution (500 μL/min) and non-solvent content (60%). The purification yield increases with the electric potential difference and reaches a maximum when the electric potential is over 200 V, which indicates that the QDs with electrophoretic mobility lower than the critical mobility do not adhere onto the electrode by electrophoresis.

Overall, this simple theoretical model could successfully predict the purification yield and it could constitute a suitable guideline for the optimization of the purification process.

### Scalability of electrophoretic purification process

**Figure 5:**
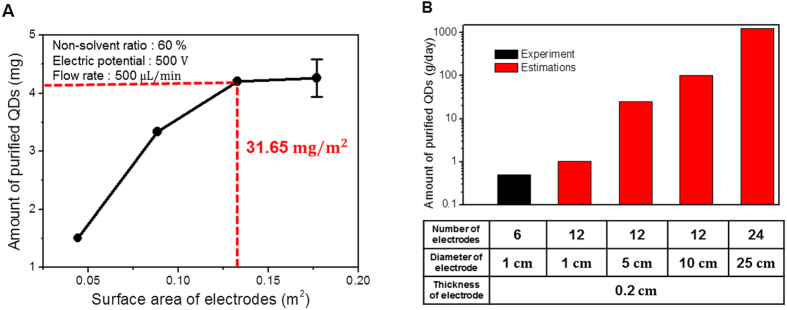
Estimation for large-scale purification. (**A**) Electrodes surface area requirement for the purification of a target amount of QDs. (**B**) Experimental (black) and estimated (red) amount of purified QDs per day with respect to the number and diameter of the electrodes.

The correct ratio of the surface areas of the electrodes to the amount of purified QDs is an important factor. When the surface area of the electrode is insufficient for the adhering QDs, not all QDs with sufficient electrophoretic mobility can attach onto the electrodes while the solution flows through the device. The specific surface area of a porous electrode was measured using the Brunauer, Emmett, and Teller method and was used to calculate the actual surface area of the electrode by multiplying with the mass of the electrode. To determine the required electrode surface area for the target amount of purified QDs, experiments were performed with various surface areas (0.044, 0.088, 0.133, and 0.177 m^2^) for a specific amount of injected QDs without changing all the other conditions (non-solvent content 60%, electric potential difference 500 V, and flow rate 500 μL/min). The results show that when the surface area of the electrode is over 0.133 m^2^, the amount of purified QDs reaches a plateau at 4.20 mg and no longer changes (Fig. 5A). Therefore, the required surface area for the target amount of purified QDs was simply calculated to be 31.6 m^2^/g.

We also investigated the possibility of scaling up the proposed process for use in industrial sites. By increasing the area of the porous electrodes, the purifying capacity could be increased without reducing the purification yield. Stacking of electrode layers could be another approach to scale up the method. Figure 5B shows the estimated amount of purified QDs per day with respect to different numbers and diameters of electrodes. The use of 24 electrodes with 25 cm diameter could provide over 1 kg of purified QDs per day.

## Conclusions

We have demonstrated a new continuous process for the large-scale purification of QDs using porous electrodes in a macroscopic flow channel. The novelty of this device is that the directions of the electric field and the fluid flow are parallel to effectively capture the QDs on the electrodes. Additionally, the proposed process includes a washing step to improve the quality of the final products without any additional cost. The device was used to purify the crude solutions of two different QDs (CdSe and PbS). Compared with the conventional purification method, the proposed technique achieved comparable purification quality and lower time and solvent consumption. Moreover, a simple analytical model was developed based on the QD travel time to describe the purification process. Both the experimental and theoretical results showed that the non-solvent content in the infused QD dispersion, the electric field strength in the flow channel, and the flow rate of the QD dispersion affect the migration of QDs in the flow channel and the purification yield. As the non-solvent content increases, the electrophoretic mobility of the QDs and the purification yield increase. The purification yield increases as the electric field strength increases or the flow rate decreases and reaches a maximum. We achieved a yield above 80%, which is much higher than that obtained by an earlier continuous process (approximately 60%)^25^. This yield can be further improved by increasing the absolute magnitude of the QD mobility by adding solvents with high dielectric constants. The continuous purification process could be scaled up using relatively simple techniques, such as increasing the number of electrodes and the surface area of electrodes. We estimated that over 1 kg/day of purified QDs can be obtained using 24 electrodes with 25 cm channel diameter. The present work shows a new possibility for the mass production and industrial use of high-quality QDs.

## Materials and Methods

### Chemicals

All the following chemicals were used as received: cadmium oxide (CdO, 99.99%, Aldrich), lead (II) oxide (PbO, 99.999%, Alfa Aesar), selenium shot (Se, 99.999%, Alfa Aesar), bis(trimethylsilyl)sulfide ((TMS)_2_S, synthesis grade, Aldrich), oleic acid (OA, technical grade and 99% from Aldrich and Alfa Aesar, respectively), tetrachloroethylene (TCE, 99%, Aldrich), and 1-octadecene (ODE, technical grade, Aldrich).

### Synthesis of CdSe QDs

CdSe QDs were synthesized following a previously reported method with some modifications^32^. 0.255 g of CdO, 3.1 mL of OA (technical grade), and 30 mL of ODE were loaded in a three-neck flask and degassed at 100 °C for 1 h. After degassing, the temperature was increased to 230 °C under N_2_ flow and maintained at that temperature for 15 min to ensure the complete conversion of Cd-oleate. Subsequently, a mixture of TOPSe (0.3 mL of 2 M TOPSe) and ODE (2.5 mL) was rapidly injected. Eight minutes after the first injection, 0.3 mL of 2 M TOPSe was added in a dropwise fashion. For the further growth of QDs, the dropwise injection step were repeated twice (0.3 mL of 2 M TOPSe for each injection) with a reaction time of 5 min for each injection. Finally, the three-neck flask was cooled by removing the heating mantle.

### Synthesis of PbS QDs

PbS QDs were synthesized using a previously reported method with some modifications^33^. 0.45 g of PbO, 1.5 mL of OA (99%), and 10 mL of ODE were placed in a three-neck flask. The mixture was degassed at 100 °C for 1 h. Then, the temperature was increased to 110 °C under N_2_ flow and 210 μL of (TMS)_2_S in 4 mL of ODE was rapidly injected. The heating mantle was removed immediately after the injection to cool the flask.

### Conventional Purification Method

For CdSe QDs, 15 mL of ethanol and 15 mL of acetone were added to 5 mL of CdSe QD crude solution and then centrifuged for 10 min at 6000 rpm. The supernatant was discarded and the precipitate was redispersed in 2 mL of toluene. This solution was centrifuged again for 10 min at 6000 rpm with the addition of 10 mL of ethanol and 10 mL of acetone and then redispersed in toluene, TCE, or benzene-d_6_ for further characterization.

For the PbS QDs, 15 mL of ethanol and 15 mL of acetone were added to 5 mL of PbS QD crude solution and then centrifuged for 10 min at 6000 rpm. The supernatant was discarded and the precipitate was redispersed in 5 mL of toluene. After adding 10 mL of ethanol and 10 mL of acetone, the solution was centrifuged for 10 min at 6000 rpm. The PbS QDs were redispersed in toluene, TCE, or benzene-d_6_ for further characterization.

### Fabrication of Electrophoretic Purification Device

The purification device is an assembly of 4-mm-thick unit layers made of polytetrafluoroethylene, which is chemically resistant to organic solvents. Each layer has a 10-mm-diameter hole that forms the flow channel. We fabricated the unit layers using a conventional machining process. A square 2-mm-thick porous nickel electrode was inserted in each layer perpendicular to the flow, as shown in Fig. 1. Polytetrafluoroethylene O-rings were inserted between adjacent layers to prevent leakages.

### Electrophoretic Purification Process

The as-prepared QD solutions were infused into the fabricated device using a syringe pump (KDS LEGATO 210, KD Scientific). The flow rate of the as-prepared QD solution ranged from 100 μL/min to 50 mL/min. An electric potential difference of 50–500 V was applied to the porous electrodes using a DC power supply (DADP-5001R, DAU NANOTEK) to generate the electrophoretic migration of the QDs in the device. After the QDs were captured by the porous electrodes, washing solutions were injected into the device at 1 mL/min. Finally, clean solvent was infused into the device at 1 mL/min and the purified QDs on the surface of the porous electrodes were redispersed into the clean solvent.

### Measurement of QD properties

The absorption spectra were obtained using a UV/Vis/NIR spectrophotometer (UV 3600, Shimadzu) and samples were dispersed in TCE. The emission spectra were recorded with a Horiba Fluorolog spectrometer at room temperature using a xenon lamp as the excitation source. NMR data were collected using an Agilent 400 MHz 54 mm DD2. The electrophoretic mobility of the QDs was measured by a zeta-potential and particle-size analyzer (ELSZ 2000, Otsuka Electronics Co.).

## Additional Information

**How to cite this article:** Lim, H. *et al*. Continuous Purification of Colloidal Quantum Dots in Large-Scale Using Porous Electrodes in Flow Channel. *Sci. Rep.* **7**, 43581; doi: 10.1038/srep43581 (2017).

**Publisher's note:** Springer Nature remains neutral with regard to jurisdictional claims in published maps and institutional affiliations.

## Supplementary Material

Supporting Information
