# Supplementary material for: Continuous Purification of Colloidal Quantum Dots in Large-Scale Using Porous Electrodes in Flow Channel
Source: Sci Rep. 2017 Feb 27;7:43581. doi: 10.1038/srep43581 (PMC5327487; doi:10.1038/srep43581)
Supplement: Supporting Information [file srep43581-s1.doc]

Supplementary Information

**Continuous Purification of Colloidal Quantum Dots in Large-Scale Using Porous Electrodes in Flow Channel**

Hosub Lim^†,‡^, Ju Young Woo^†,∥^, Doh C. Lee^∥^, Jinkee Lee*^,‡^, Sohee Jeong*^,†, §^, and Duckjong Kim*^,†^

^†^ Department of Nano Mechanics, Korea Institute of Machinery and Materials (KIMM), Daejeon 34103, Republic of Korea

^‡^ School of Mechanical Engineering, Sungkyunkwan University, Suwon, Gyeonggi-do 16419, Republic of Korea

^∥^Department of Chemical and Biomolecular Engineering (BK+ Program), KAIST Institute for the Nanocentury, Korea Advanced Institute of Science and Technology (KAIST), Daejeon 34141, Republic of Korea

^§^University of Science and Technology (UST), 217 Gajeong-ro, Daejeon 34113, Republic of Korea

*Corresponding Author’ emails: [lee.jinkee@skku.edu](mailto:lee.jinkee@skku.edu), [sjeong@kimm.re.kr](mailto:sjeong@kimm.re.kr) and [dkim@kimm.re.kr](mailto:dkim@kimm.re.kr)


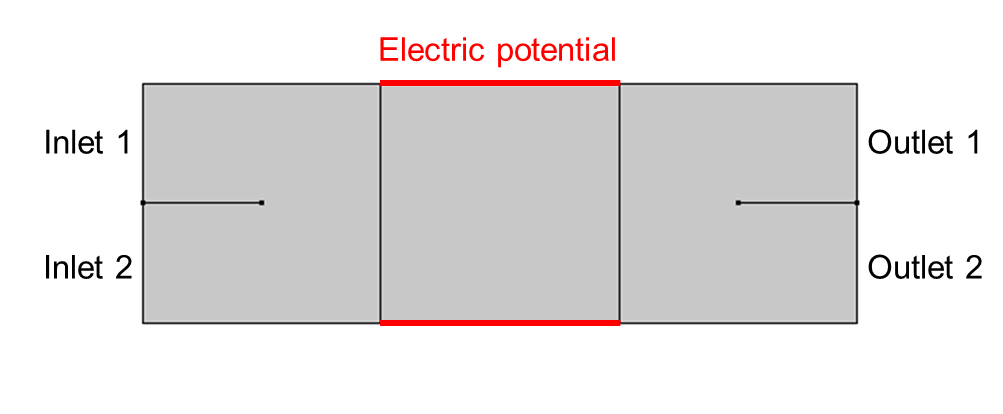


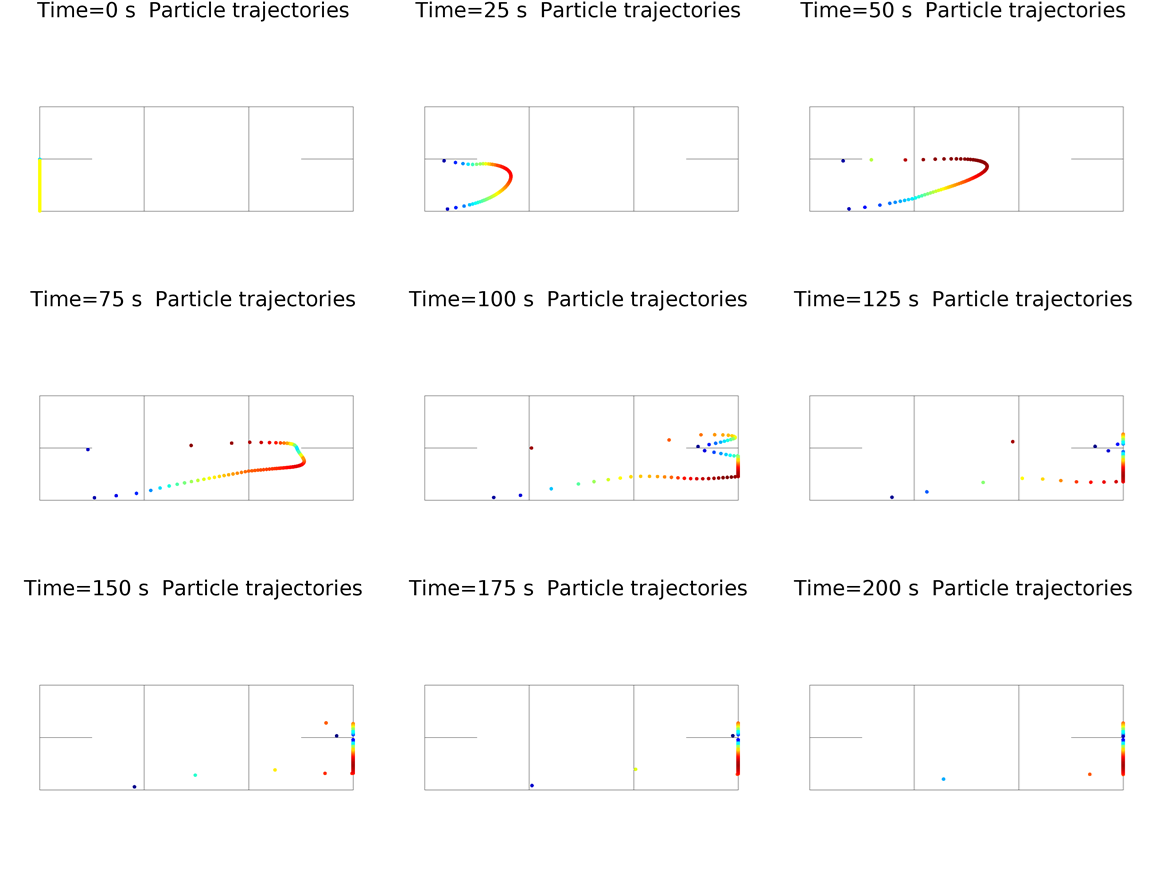


**Figure S1.** Results of a particle tracing simulation (COMSOL) considering the direction of the flow to be perpendicular to the electric field. The red lines show the electrode. The particles migrate toward the upper channel owing to the electric field. The flow velocity was 5×10^-5^ m/s, the electrical potential was 500 V, and the density, radius, and charge number of the particle were 5.82 g/m^3^, 3 nm, and -1, respectively.


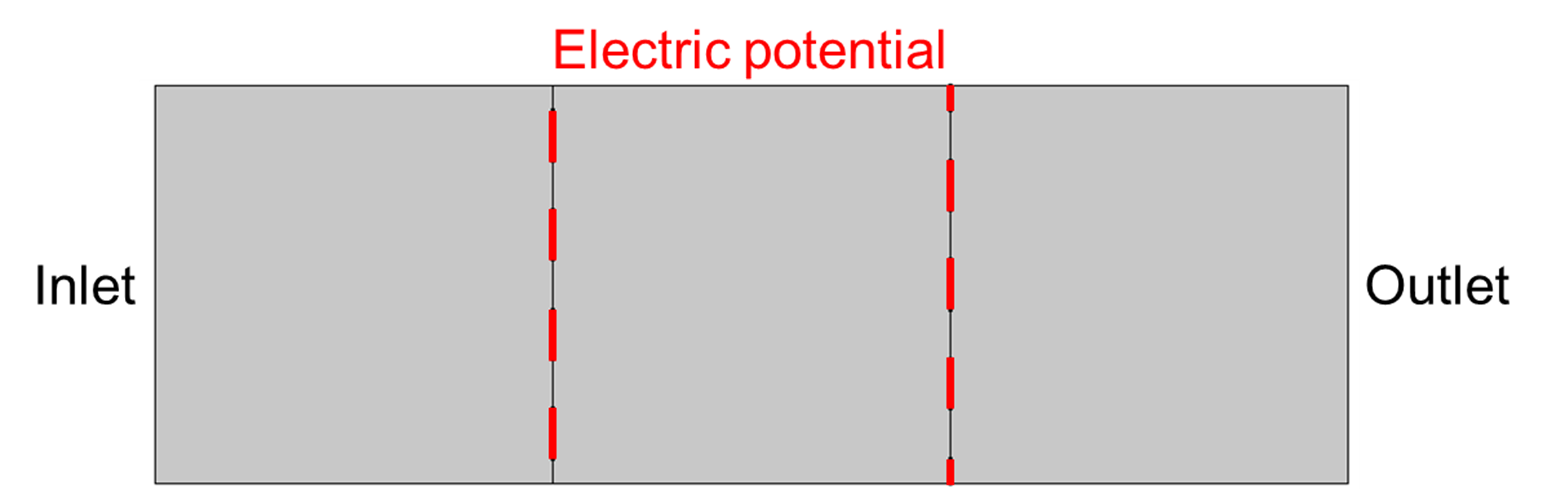


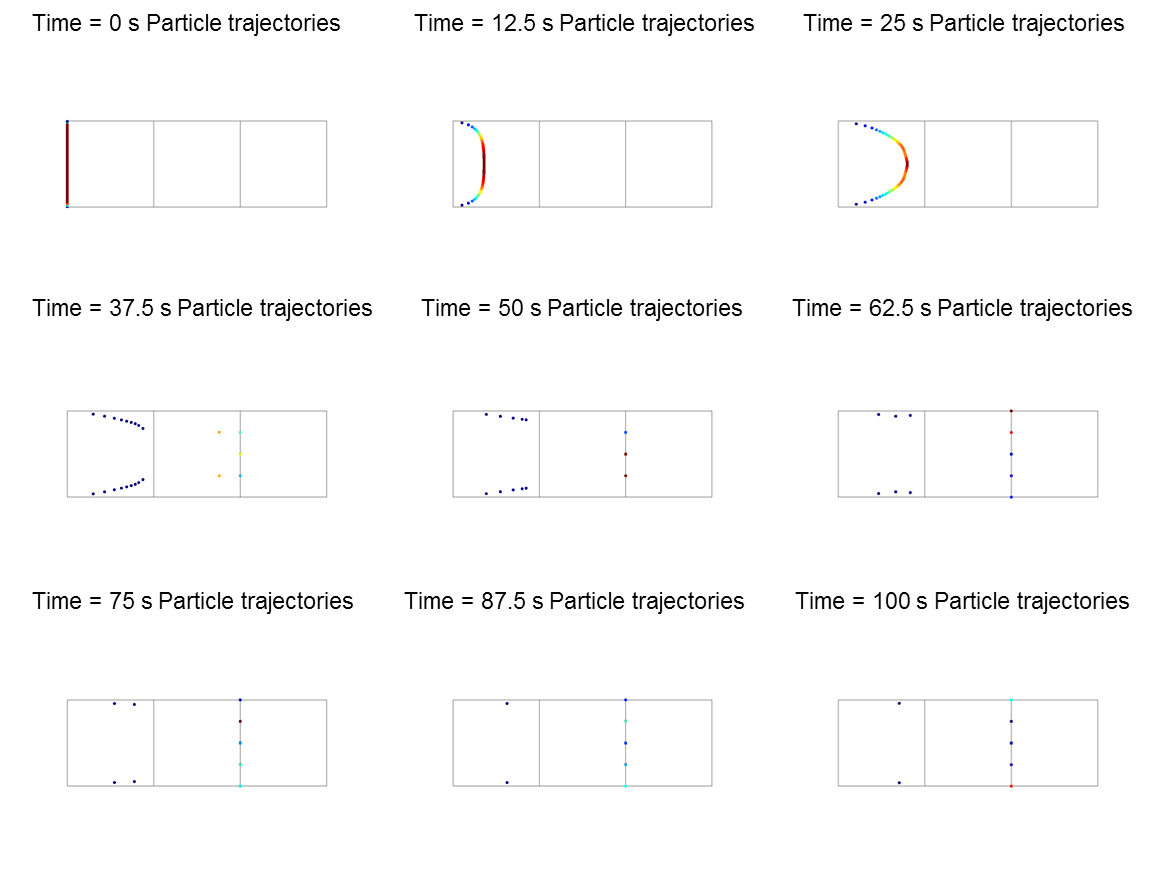


**Figure S2.** Results of a particle tracing simulation (COMSOL) considering the direction of the flow to be parallel to the electric field. The red lines show the electrode. The particles adhere onto the electrode owing to the electric field. The flow velocity was 5×10^-5^ m/s, the electrical potential was 500 V, and the density, radius, and charge number of the particle were 5.82 g/m^3^, 3 nm, and -1, respectively.


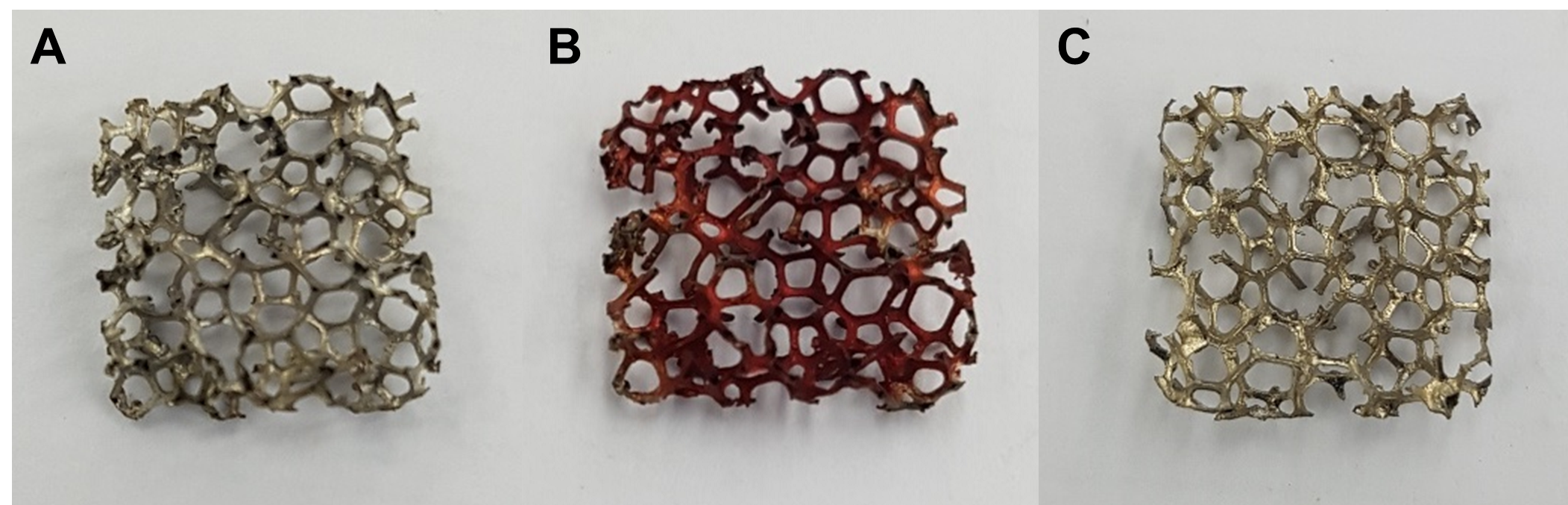


Figure S3. The images of electrode (A) in the absence of the electric field, and in the presence of the electric field with (B) positive and (C) negative polarity, respectively. Experimental conditions were **maintained stable, with 60% non-solvent contents, 500 V electrical potential and 500 μL/min flow rate.**


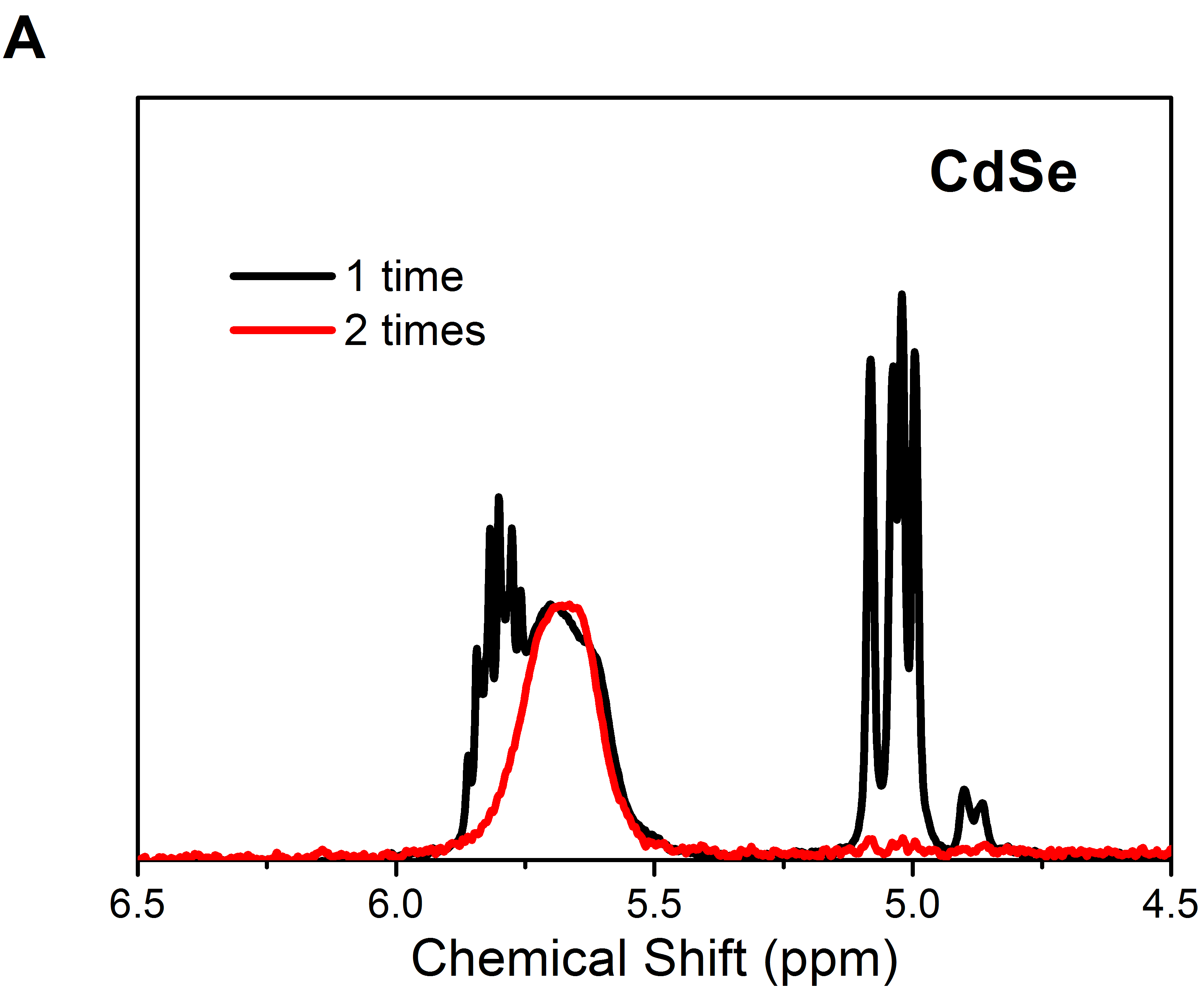


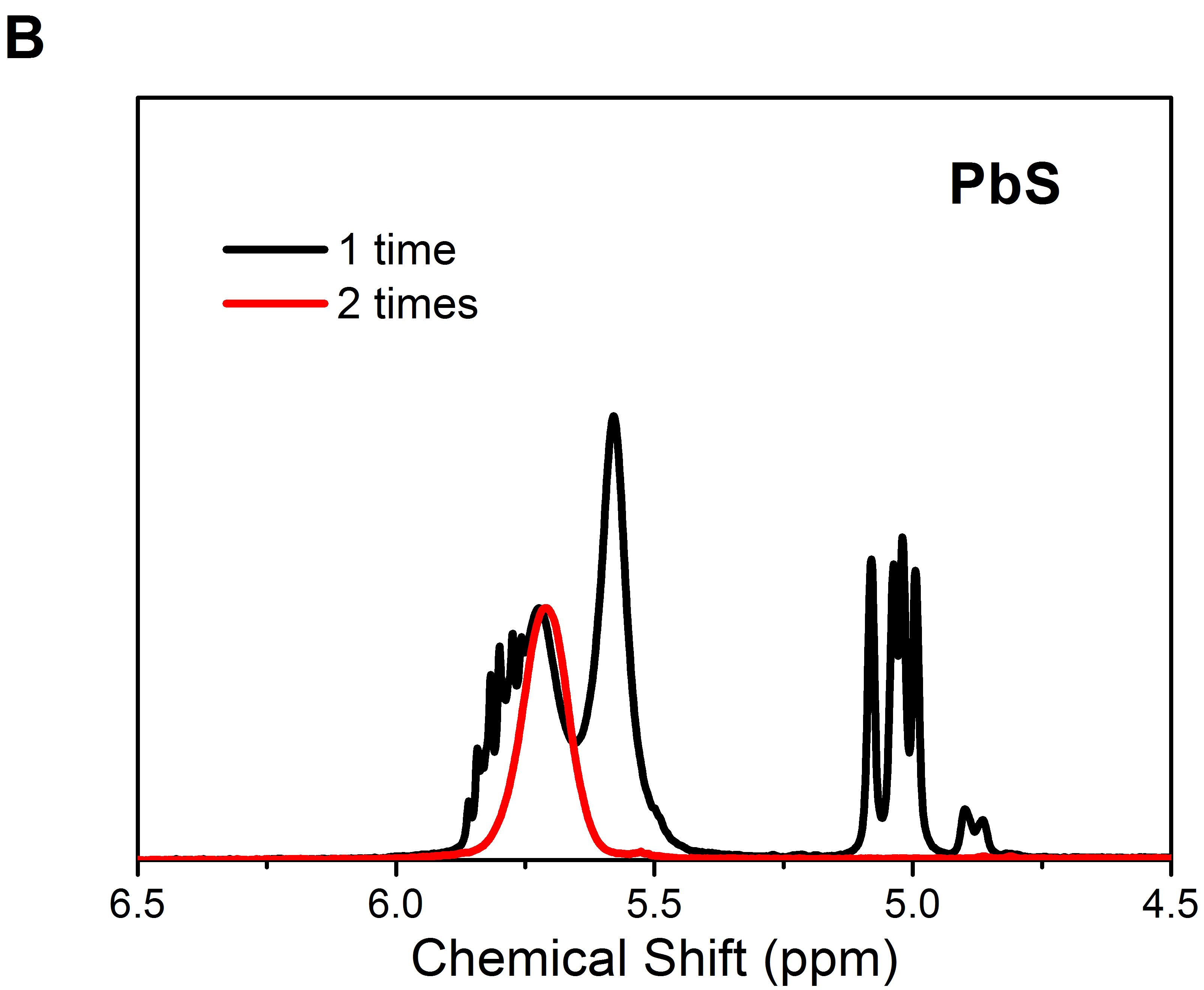


**Figure S4.** ^1^H NMR spectra of (A) CdSe QDs and (B) PbS QDs for different repetition numbers of the precipitation–redispersion method.


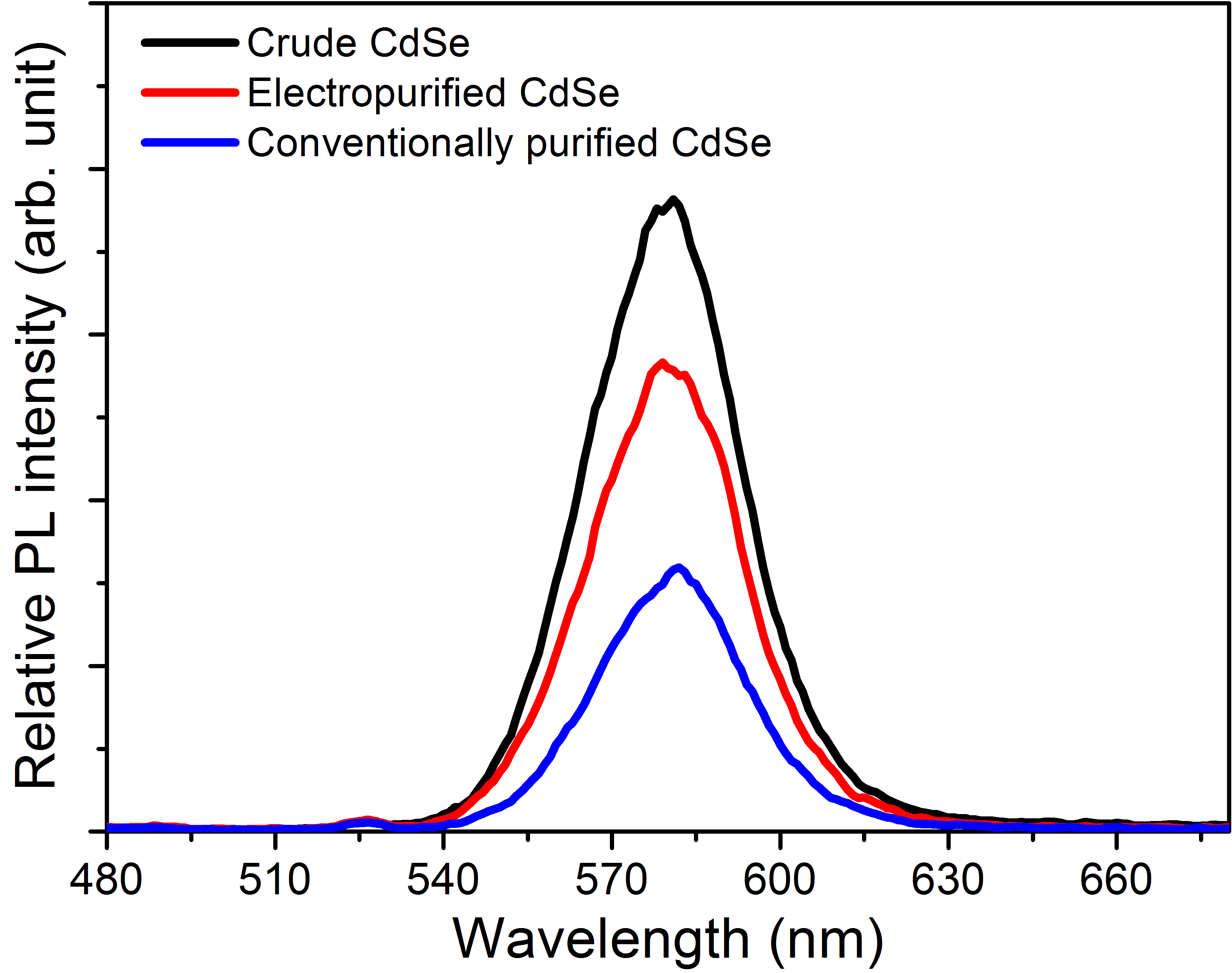


**Figure S5.** Relative PL spectra of crude, electropurified, and conventionally purified CdSe.


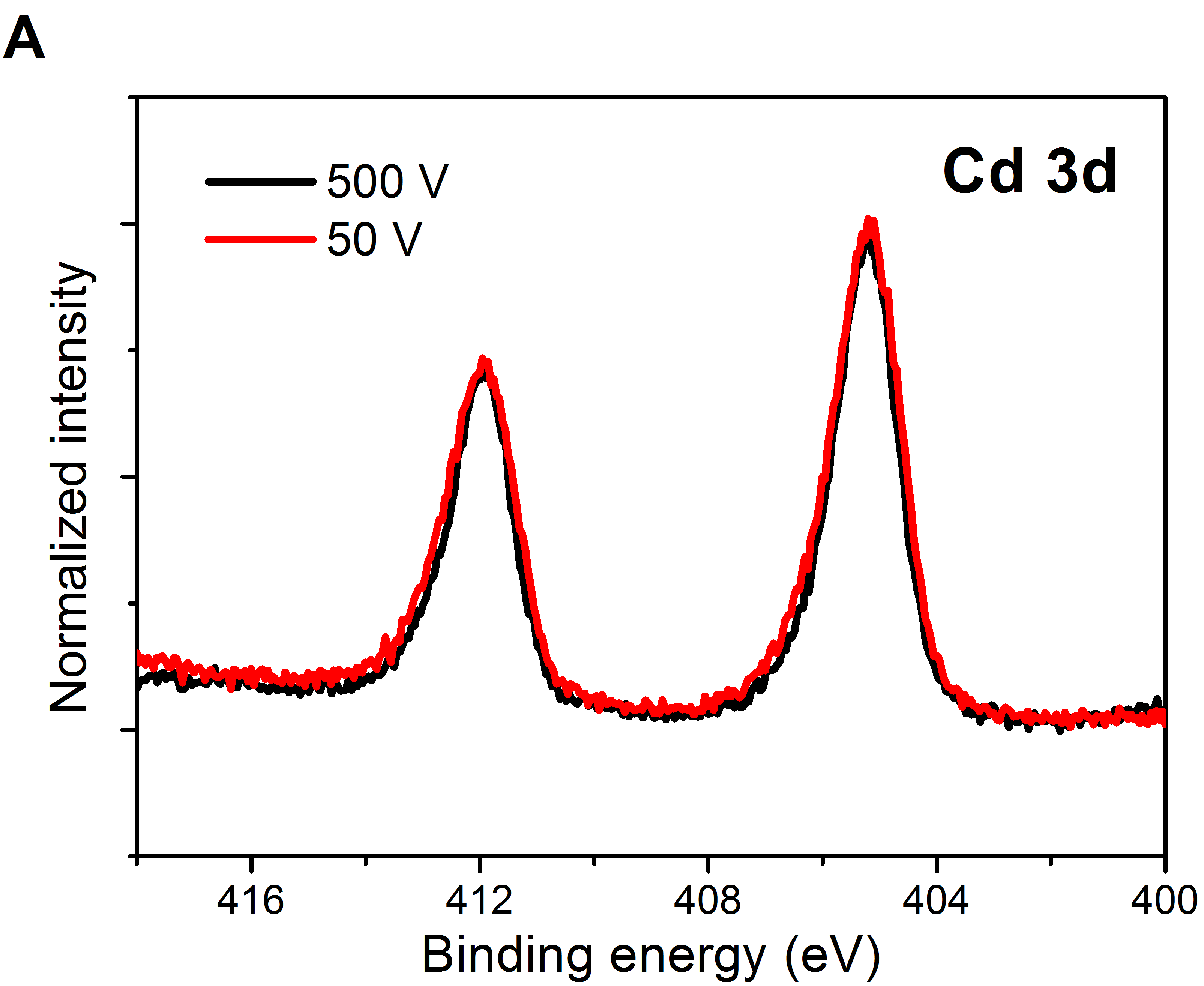


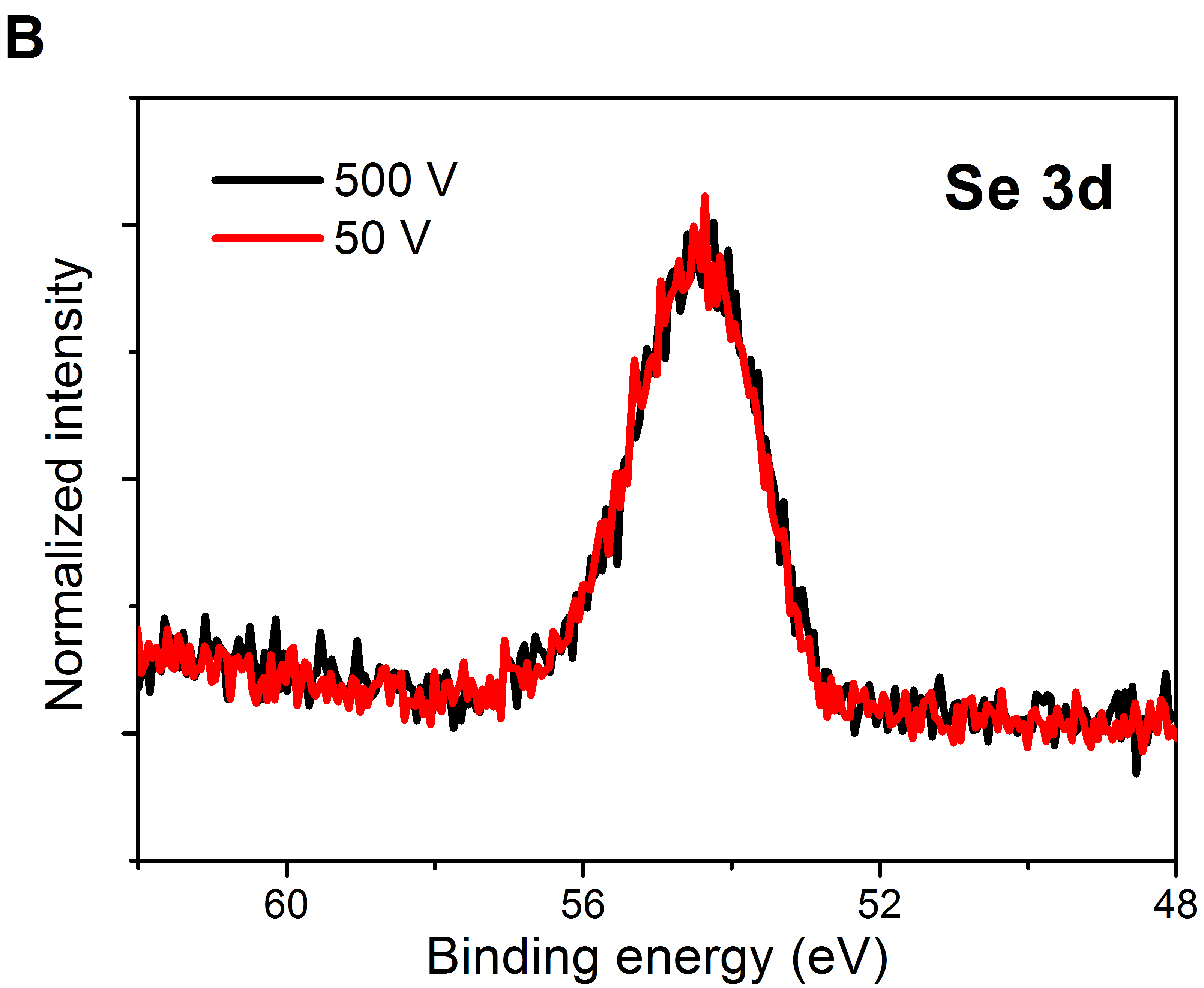


**Figure S6.** (A) Cd 3d and (B) Se 3d XPS spectra of electropurified CdSe.


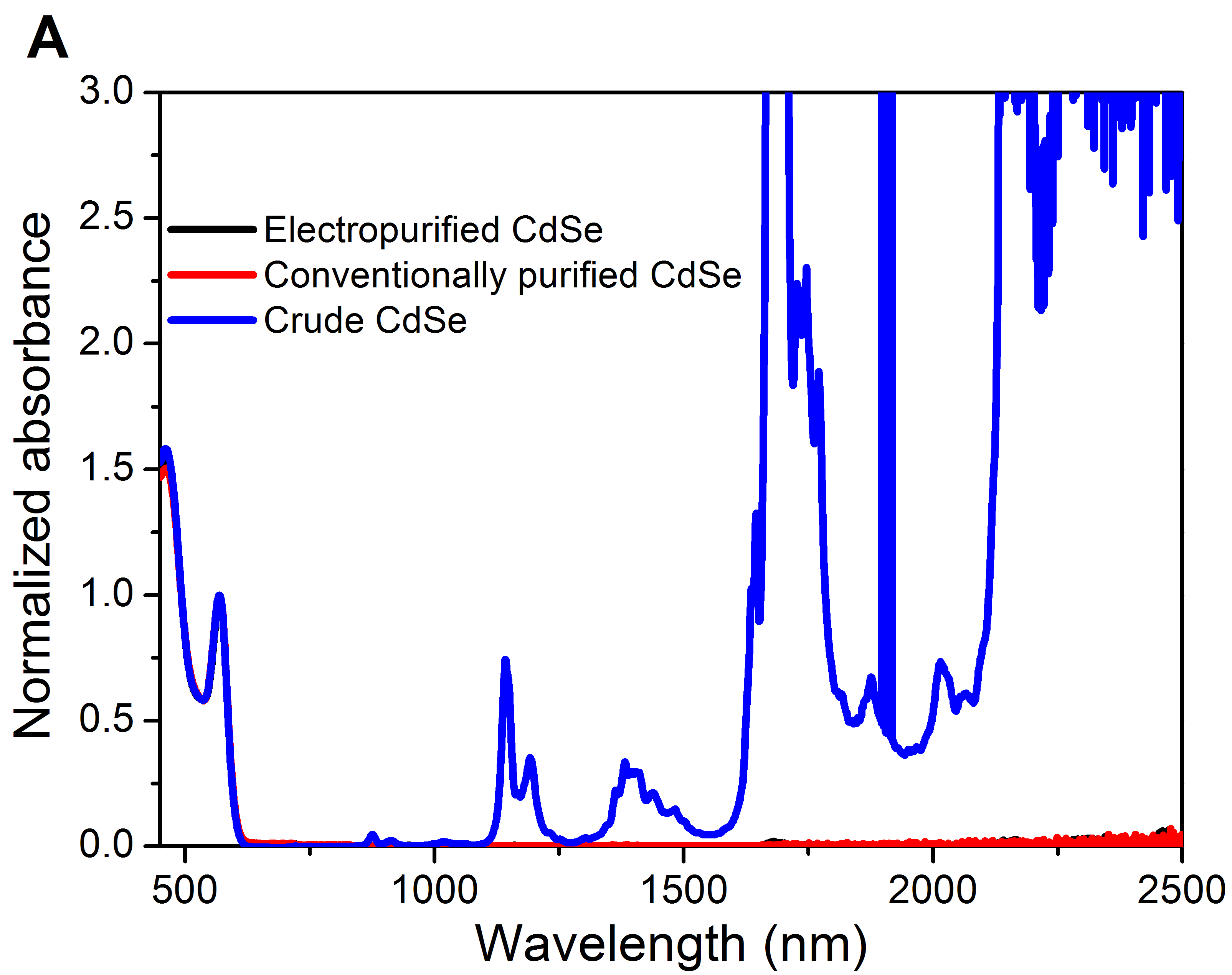


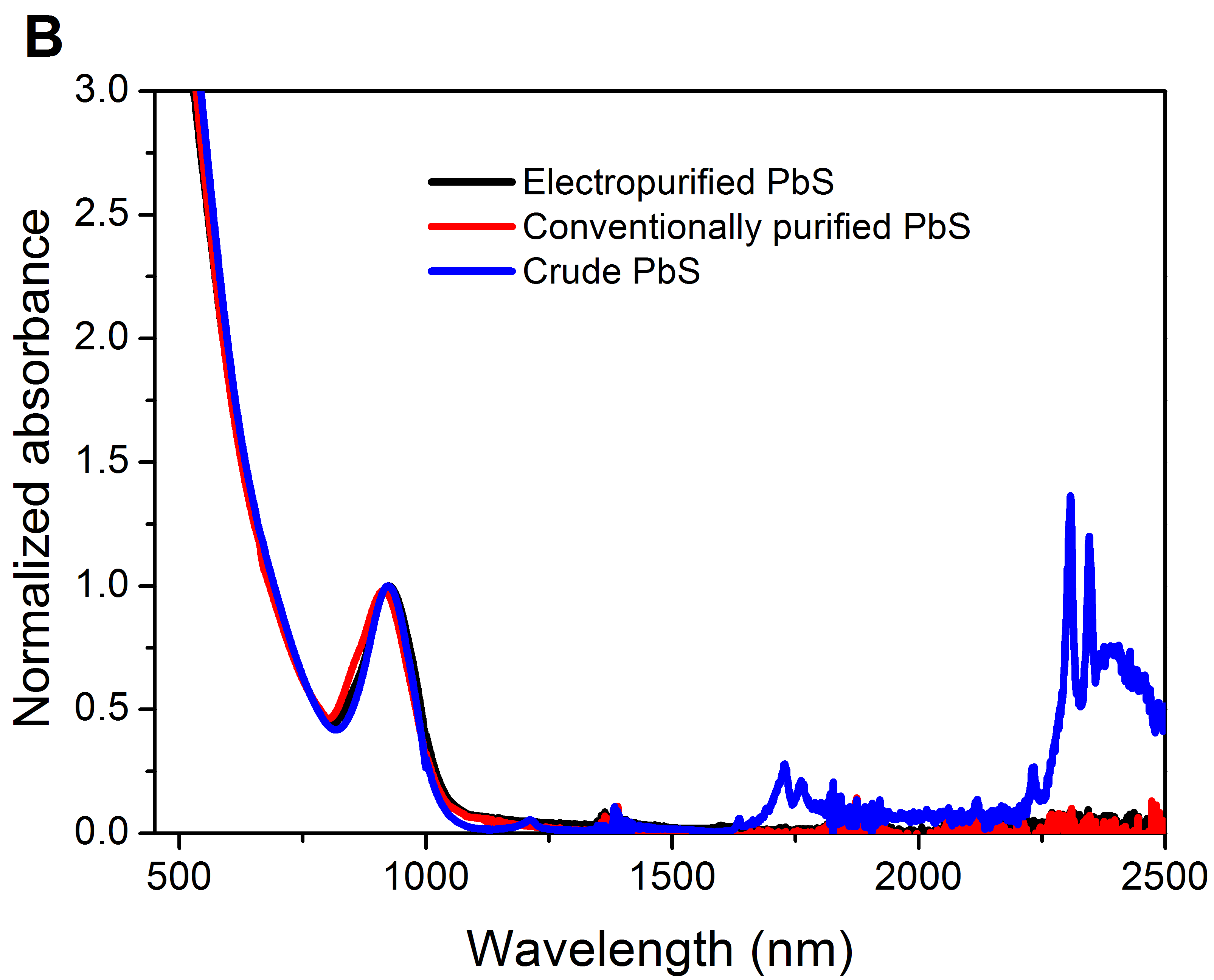


**Figure S7.** (A) The absorption of electropurified CdSe QDs compared to that of unpurified and conventionally purified CdSe QDs. Typical impurities of the crude QD solution include free oleate, metal oleate, and ODE, which are observed in the NIR region. The optical density of the electrophoretically and conventionally purified QDs is almost zero in the NIR region. (B) The absorption of electropurified, conventionally purified, and crude PbS QDs, which display similar results to those of CdSe QDs.


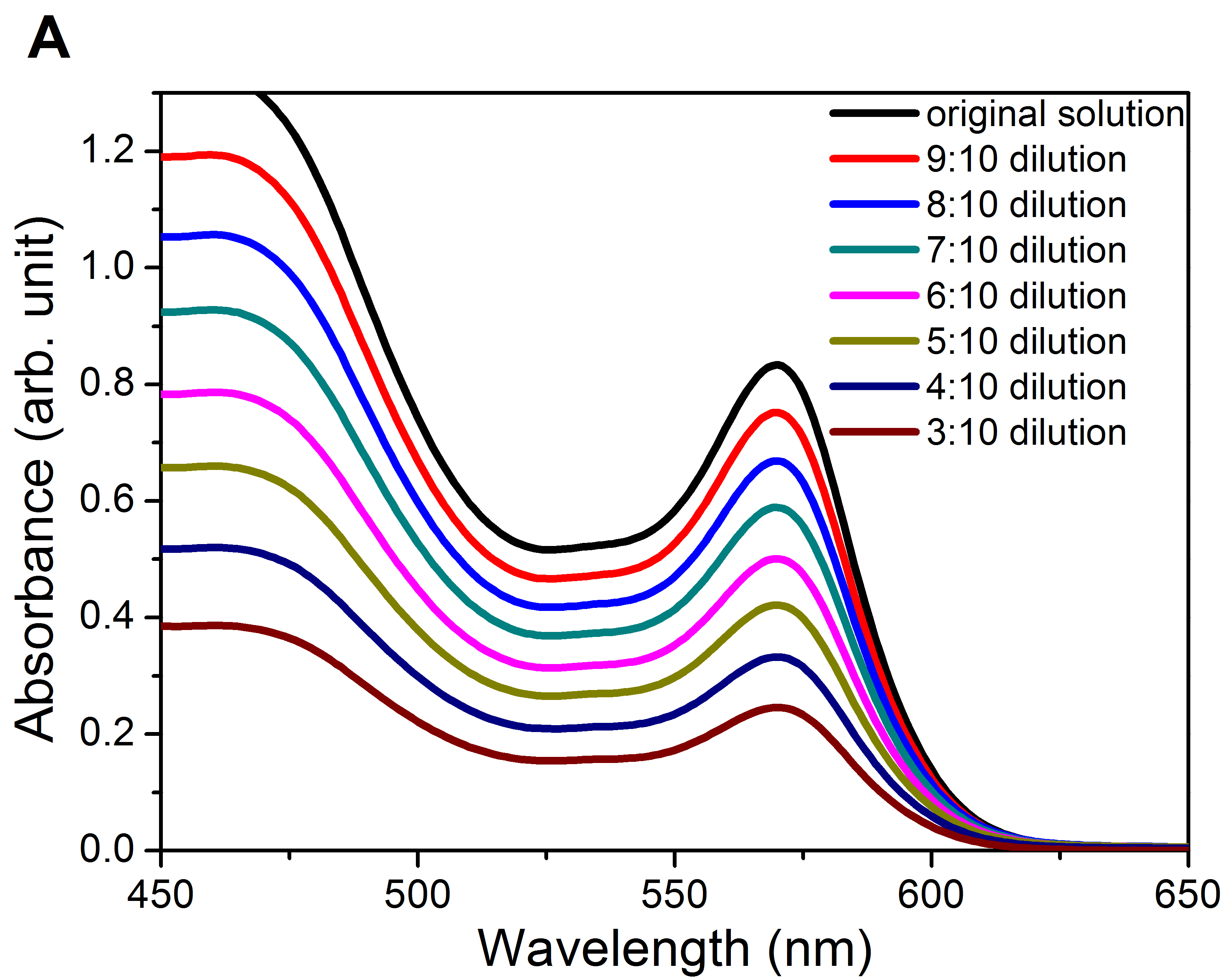


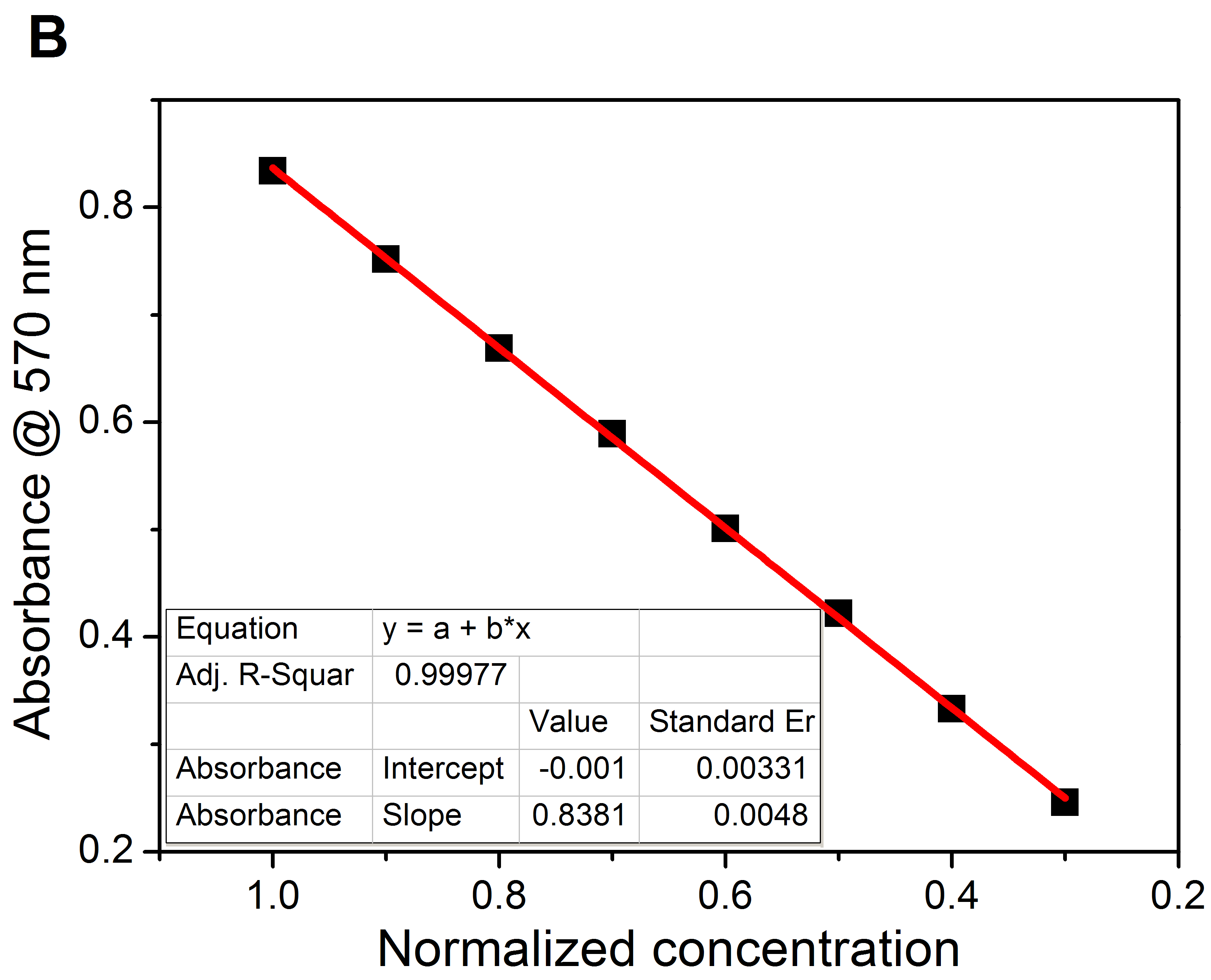


**Figure S8.** (A) The absorbance at 570 nm of CdSe QD solutions with various concentrations. (B) The linear relationship between QD concentration and absorbance confirming the Beer-Lambert law.
